# Prenatal Diagnosis of 17p13.1p13.3 Duplication

**DOI:** 10.1155/2012/840538

**Published:** 2012-10-17

**Authors:** Kirsi Kiiski, Tiiu Roovere, Riina Zordania, Harriet von Koskull, Nina Horelli-Kuitunen

**Affiliations:** ^1^Department of Genetics, United Medix Laboratories Ltd., 00380 Helsinki, Finland; ^2^Department of Clinical Genetics, Helsinki University Central Hospital, 00290 Helsinki, Finland; ^3^Nova Vita Kliinik, 11136 Tallinn, Estonia; ^4^Department of Genetics, Tartu University Hospital, 50406 Tartu, Estonia

## Abstract

We present here the first prenatal diagnosis of 17p13.1p13.3 duplication. 17p13.3 duplication has recently been defined as a new distinctive syndrome with several diagnosed patients. In the current case prenatal chromosome analysis (G-banding) performed on cultured amniocytes revealed additional material in chromosome 19p. This was further defined as a chromosome 17p13.1p13.3 duplication by FISH and genomic microarray analysis (GMA). In addition Prenatal BACs-on-Beads (PN_BoBs) assay was performed, which detected the duplication clearly. This enables rapid prenatal diagnosis of the duplication for this family in the future.

## 1. Introduction

Duplication of a region on chromosome 17p13.3 has emerged as a new distinctive syndrome (MIM #613215). Phenotypic features include intrauterine growth retardation (IUGR), psychomotor delay, hypotonia, craniofacial dysmorphism, and subtle hand/foot malformations [1–4]. Some variation is known to occur in the breakpoints of the duplicated region and consequently in the gene content as well. This variation is reflected to the slight variations in phenotype. Deletion of the same region on chromosome 17p13.3 causes the Miller-Dieker lissencephaly syndrome (MIM #247200). We have focused on the diagnosis of this syndrome because of a prenatal case. 

## 2. Case Presentation

**Figure 1:**
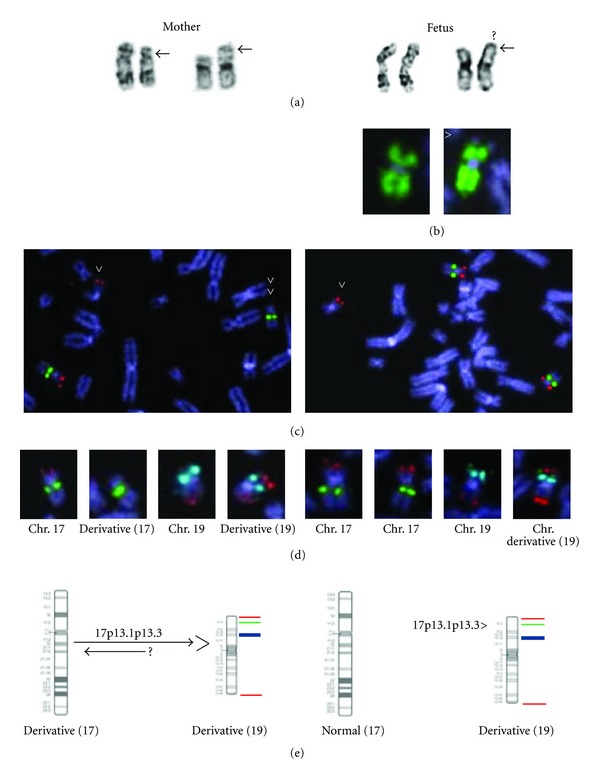
Chromosome and FISH results. On the right side fetus (amciocytes) and left side mother (lymphocytes). All FISH probes were from Vysis-Abbott. (a) Karyogram images of chromosomes 17 and 19. Both the fetus and the mother have additional material in chromosome 19p. The fetus has normal chromosomes 17 but the mother has a deletion in one chromosome 17p indicating that she is a carrier of a potentially balanced translocation. (b) Chromosome painting with a wcp19 probe shows a small unpainted terminal region on chromosome 19p (arrowhead). (c) MDS microdeletion specific probe (17p13.3-red/LIS1-gene and 17q21-green/RARA-gene) for the fetus and the mother. An extra red signal is seen on the der(19) chromosome both in the fetus and in the mother (1x arrowhead). In the mother the Miller Dieker syndrome specific probe shows MDS probe on der(19) and the control probe on der(17). 2x arrowheads point to the der(17) of the mother that lacks the MDS probe signal. (d) Simultaneous hybridization with MDS probe and subtelomere probe set for chromosome 19 (19p/19p13/19q, ToTelVysion, Vysis-Abbott) shows the order of these probes indicating that the MDS region has been translocated/inserted distal to the 19p subtelomere region in the der(19). (Mixtures 8 and 14 were applied from ToTelVysion probe kit, which are specific for chromosome 17p subtel/17cep and 19p subtel/19p13/19q, respectively). (e) Ideogram images of chromosomes 17 and 19. FISH probes are marked from p arm to q arm with different colour (17p13.3/red-19ptel/green-19p13/aqua-19qtel/red).

A 23-year-old primigravida had amniocentesis because of increased trisomy risk indicated by first trimester trisomy screening (first trimester combined risk; T21 = 1 : 72, double test T18 = 1 : 50, NT = 6.5 mm). In addition IUGR was detected by ultrasound. The parents were both Caucasians, healthy and nonconsanguineous with unremarkable family history. Amniocentesis was performed at week 15 of gestation. Prenatal chromosome analysis (G-banding) performed on cultured amniocytes (*in situ* and long term) indicated the presence of additional material in chromosome 19 of the fetus. The finding was confirmed and specified by FISH using a 19p subtelomeric probe and whole chromosome 19 painting probes. The chromosome and FISH studies are shown in Figure 1. Chromosome analysis of the parents showed that the mother was a balanced translocation carrier having a translocation between chromosomes 17 and 19. The fetus had inherited the derivative chromosome 19 but a normal chromosome17, therefore having partial 17p trisomy. The father had a normal karyotype. 

The cytogenetic structure of the derivative chromosome 19 of the fetus and the translocation chromosomes (17 and 19) of the mother were further characterized by different FISH probes and probe combinations from ToTelVysion probe set (Vysis-Abbott), wcp19 (Vysis-Abbott), Miller-Dieker microdeletion syndrome specific probe (Vysis-Abbott). Both the subtelomeric region of 17p and 17p13.3 (Miller-Dieker probe) were shown to reside on the derivative chromosome 19 of the fetus and the karyotype was thus interpreted as 46, XX, der(19)t(19; 17)(p13.3; p12p13.3)mat. A translocation, comprising solely of a one directional translocation or insertion of a segment of one chromosome (17p) into another chromosome (19p) would be rare. However, any reciprocal event could not be shown. 

After genetic counselling the parents decided to terminate the pregnancy. Termination of pregnancy was performed at week 20 of gestation. Even though the most typical characteristics for this syndrome such as developmental and psychomotor delay appear after birth, some of the phenotypic features of the syndrome could be identified. These were similar to previously described patients, including growth retardation, a prominent forehead and occiput, bitemporal hollowing, deformed earlobes, micrognathia, short sternum, and arachnodactyly. 

**Figure 2:**
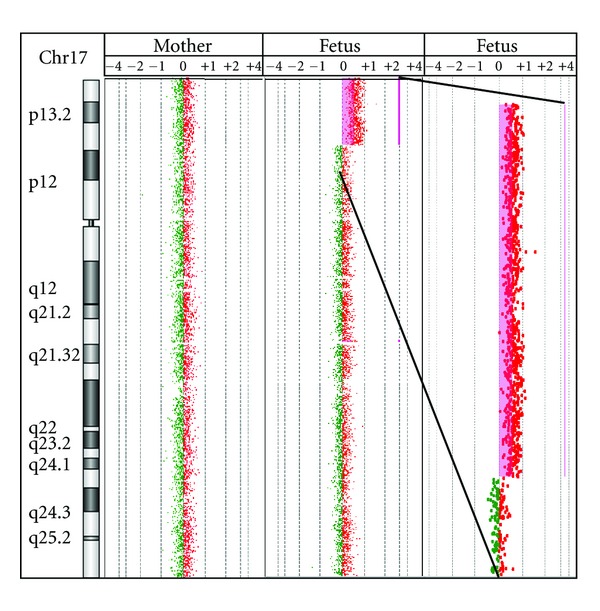
GMA results. The GMA results show a duplication of 17p13.1p13.3 of the fetus and a normal result of the mother.

To precisely define the breakpoints of the duplicated 17p region of the fetus, genomic microarray (GMA) was performed on DNA extracted from fetal skin fibroblasts and maternal blood (Agilent, Human 180 K array, Hg18, Agilent Technologies, Santa Clara, CA, USA). GMA of the fetus revealed a 10.7 Mb duplication in chromosome 17p (Figure 2) but no deletion in chromosome 19 could be identified. No other significant copy number variations were detected either. The molecular karyotype of the fetus was thus: arr17p13.3p13.1(51,885–10,744,346)x3 mat. The mother had a normal molecular karyotype, which supported the fact that she was a carrier of a balanced chromosomal translocation.

**Figure 3:**
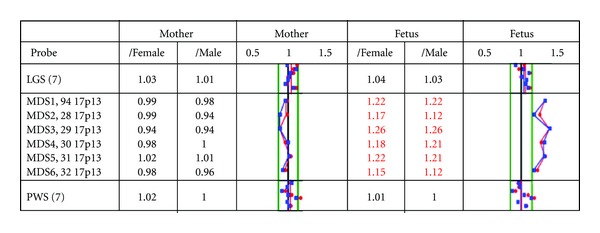
PN_BoBs results. The Prenatal BACs-on-Beads-assay (PN_BoBs) results show a duplication of all six probes on the Miller-Dieker syndrome (MDS) region of the fetus and a normal result of the mother.

In addition to these analyses a new bead-based multiplex assay Prenatal BACs-on-Beads (PN_BoBs) (PerkinElmer Wallac OY, Turku, Finland) was performed to validate the PN_BoBs method. PN_BoBs was done on DNA extracted from fetal skin and maternal lymphocytes. The PN_BoBs utilizes Luminex xMAP technology (Luminex Corporation, Austin, TX, USA) and it is a rapid detection method for common trisomies (13, 18, 21, X and Y) in addition to nine microdeletions targeting known microdeletion syndromes [5–8]. We wanted to test the accuracy of the PN_BoBs assay for this 17.p13.1p13.3 duplication. The test showed clearly a duplication of the Miller-Dieker region (MDS/17p13.3) in the fetal DNA and a normal result in the maternal sample (Figure 3). 

## 3. Discussion

This prenatal 17p13.1p13.3 duplication was originally detected by conventional karyotyping and FISH, but the GMA analysis enabled the exact detection of the break points of the duplicated region. This helps to further characterize the 17p13.1p13.3 duplication syndrome and gives exact information about the gene contents of the duplicated region. This is important as especially the presence or absence of the genes *PAFAH1B1 *encoding LIS1, *YWHAE* encoding 14-3-3*ε*, and *CRK* encoding Crk seem to define the phenotype of the syndrome. 

The overexpression of the *PAFAH1B1* gene, encoding LIS1, has been shown to affect brain development by causing migrational defects [1, 9]. The duplicated region also covered the genes *YWHAE *and *CRK. YWHAE* encodes 14-3-3*ε*, whose dosage alterations are suggested to affect the neuronal network development and maturation. *YWHAE* has also been stated as an attractive candidate gene for autism spectrum disorders and developmental delay when duplicated [1, 3]. Crk for one is known to interact with signal pathways involved in brain and limb development [3, 10].

The 17p13.3 duplications have been divided into two classes [3]. Class I duplications involve the *YWHAE* but not *PAFAH1B1* gene and the phenotype includes autistic features, speech and motor delay, subtle facial, and hand/foot dysmorphisms [1, 3]. Class II duplications in turn always include *PAFAH1B1 *and may also include *YWHAE* and *CRK* genes. The phenotypic features in Class II are suggested to be milder including mild developmental and psychomotor delay [3]. It has also been hypothesized that when all of the three genes are duplicated, the phenotypic features seem to be more complex due to the interactions between the several duplicated genes involved [2]. This current case belongs to the Class II category as all of the three genes are duplicated. Based on this the phenotype might not have been severe. The fetus had, however, a considerably larger duplication (10.7 Mb) than most of the patients described so far. This usually indicates a more severe phenotype. 

In forthcoming pregnancies of this carrier woman, prenatal diagnosis can preferentially be performed using the Prenatal BACs-on-Beads assay on DNA extracted from uncultured amniotic fluid or chorionic villi. This is faster than the conventional FISH method using a Miller-Dieker microdeletion specific probe along with conventional chromosome analysis. These particular unbalanced translocation chromosomes, der(17) and der(19) of the mother, can quickly and accurately be identified by the PN_BoBs method; both the deletion and the duplication of the MDS region can be identified. The breakpoints can further be characterized using GMA analysis.

Several cases of postnatally detected 17p13.3 duplication have been published, but to the best of our knowledge, this is the first prenatal diagnosis of the 17p13.1p13.3 duplication. Defining the molecular background of the aberration enables the use of a rapid method for prenatal diagnosis in this family in the future.
